# Distributed DRL-Based Computation Offloading Scheme for Improving QoE in Edge Computing Environments

**DOI:** 10.3390/s23084166

**Published:** 2023-04-21

**Authors:** Jinho Park, Kwangsue Chung

**Affiliations:** Department of Electronics and Communications Engineering, Kwangwoon University, Seoul 01897, Republic of Korea; jhpark@cclab.kw.ac.kr

**Keywords:** reinforcement learning, interning of things, mobile edge computing, computation offloading, linear regression

## Abstract

Various edge collaboration schemes that rely on reinforcement learning (RL) have been proposed to improve the quality of experience (QoE). Deep RL (DRL) maximizes cumulative rewards through large-scale exploration and exploitation. However, the existing DRL schemes do not consider the temporal states using a fully connected layer. Moreover, they learn the offloading policy regardless of the importance of experience. They also do not learn enough because of their limited experiences in distributed environments. To solve these problems, we proposed a distributed DRL-based computation offloading scheme for improving the QoE in edge computing environments. The proposed scheme selects the offloading target by modeling the task service time and load balance. We implemented three methods to improve the learning performance. Firstly, the DRL scheme used the least absolute shrinkage and selection operator (LASSO) regression and attention layer to consider the temporal states. Secondly, we learned the optimal policy based on the importance of experience using the TD error and loss of the critic network. Finally, we adaptively shared the experience between agents, based on the strategy gradient, to solve the data sparsity problem. The simulation results showed that the proposed scheme achieved lower variation and higher rewards than the existing schemes.

## 1. Introduction

Recently, networks and Internet of Things (IoT) devices have developed rapidly [1]. These developments are providing people with a more convenient life. However, with the development of IoT applications, services such as AR, VR, and online games require very low latency to satisfy these user requirements [2,3]. IoT devices are limited because the task service time is high or the accuracy of applications is low, because they lack computing resources. Numerous researchers have proposed edge servers—also called Mobile Edge Computing (MEC)-based offloading schemes to overcome this limitation. Edge servers have sufficient computing resources and low transmission delays, so they are suitable for processing tasks [4]. In particular, the devices send created tasks to the edge server and receive the results. Despite the advantages of edge servers, when devices send several tasks to an edge server, fewer computing resources are allocated to each task; thus, the waiting times for tasks until processing are increased. In this regard, collaboration between edge servers has been proposed to improve the efficiency of task processing.

The collaboration scheme between edge servers shows high performance in processing several tasks, because it utilizes the computing resources of other edge servers [5]. However, this scheme has certain limitations. Each edge server independently determines an offloading target for a task received from a device, thereby concentrating the task on one edge server. This trend increases the task service time, and the scheme also has low resource utilization because it handles the task independently. Consequently, the collaboration scheme decreases the quality of experience (QoE) according to the high task service time and low load balance [6].

The existing collaboration scheme between edge servers determines the offloading target based on heuristic methods, such as game theory, to minimize task service times and improve load balance. The heuristic method yields optimal results in a specific environment. However, this method does not show optimal results in dynamically changing situations, such as real environments [7]. Thus, reinforcement learning (RL) is used to determine the optimal target in such environments. RL learns the optimal policy to maximize long-term rewards without prior knowledge of the system model. Moreover, deep RL (DRL), which involves the use of a deep neural network (DNN), has been introduced to process more states than RL [8].

As a state-of-the-art RL method, the deep deterministic policy gradient (DDPG) [9] shows optimal results in continuous action. A recently studied DDPG-based collaboration scheme between edge servers extracts the features for a given state using fully connected (FC) layers. However, because the FC layer does not consider temporal features, the DDPG cannot achieve a high reward. The existing RL-based schemes also learn the policy without the importance of experience. Among the randomly sampled experiences, the loss error does not reduce the loss of network, so this approach is ineffective for learning. In addition, the RL agent uses the experience generated from the local edge server. In an edge distributed environment, the RL agent has a problem of data sparsity, in which sufficient learning cannot be achieved using a small number of devices connected to the edge server.

To solve the aforementioned, we propose a distributed DRL-based computation offloading scheme for improving the QoE in edge computing environments. The proposed scheme models the task service time and load balance between edge servers to define the RL reward. Furthermore, we represent the state and action of RL and learn the optimal policy. In particular, we present three contributions to improve the reward for learning and reduce the reward variation. Firstly, we extract features using LASSO (least absolute shrinkage and selection operator)-based LAFE (feature extraction) to determine collaboration targets. LAFE uses LASSO regression to predict features for the next state. The predicted and current states are used to determine an action to improve the rewards of reinforcement learning. In RL training, an attention layer is introduced to minimize the effect of insignificant features among the extracted features. Secondly, we prioritize experiences by considering learning progress as well as TD (temporal difference) error. Based on this priority, experiences are sampled for improving training efficiency. Finally, the proposed scheme adaptively shares experiences based on the strategy gradient between agents to solve the data sparsity problem in collaborative environments between edge servers.

The remainder of this study is organized as follows: Section 2 describes the computation offloading and existing RL-based offloading schemes; Section 3 presents the problem formulation and RL structure for edge collaboration; in Section 4, the experimental results of the proposed scheme are presented; finally, the conclusions are provided in Section 5.

## 2. Background and Related Research

### 2.1. Computation Offloading

The concept of computation offloading was first proposed by M. Satyanarayann [10]. Computation offloading improves the performance of the application, and reduces the battery consumption of devices. As cloud computing gains attention, offloading schemes for computational applications to a centralized node with a considerable amount of computing resources are being studied. Sufficient computing resources, such as the cloud, are advantageous in processing tasks [11]. However, centralized computing nodes suffer from the problem of load on the backbone network when several devices transmit a task. An increase in the load on the network causes high transmission delays such that the task requirements are not satisfied.

Fog computing was proposed by Cisco in 2012; it can solve the problem mentioned above [12]. This approach can be used to overcome the limitations mentioned above. In particular, in this approach, a computing node is present between the cloud and the device, with the device having fewer computing resources than the cloud. Nevertheless, it has a low network latency. Furthermore, after the introduction of MEC by ESTI [13], the performance of computation offloading using MEC was improved [14].

### 2.2. Reinforcement Learning

Reinforcement learning learns the optimal policy according to countless explorations and exploitations to maximize the cumulative reward. As a result of sequential decision-making, a change in the state affects the learning process. RL consists of the environment, agent, state, action, and reward. Specifically, the environment represents the object to be solved using reinforcement learning. The state changes depending on the agent’s action, and the agent recognizes a state in the environment. In addition, the algorithms set the gains reward and follow to the next states through actions determined by the policy. The action explores the action space based on a trial-and-error approach to improve the policy. The reward represents the result that an agent obtains from the environment through action. The agent’s action is defined as Equation (1).

(1) $$\pi \left(a|s\right)=P[A_{t}=a|S_{t}=s]$$

a denotes the action performed by the agent, π denotes the policy, $A_{t}$ denotes the set of actions spaces obtained at time t, s denotes the state of the agent, and $S_{t}$ denotes the state space of the agent at time t. RL learns a policy by evaluating the action performed by an agent. The action evaluation is defined as Equation (2).

(2) $$Q_{\pi }\left(s,a\right)=E_{\pi }[G_{t}|S_{t}=s,A_{t}=a]$$

$G_{t}$ denotes the cumulative reward, and $E_{\pi }$ denotes the expected value based on given conditions. Therefore, RL is a method that evaluates actions according to each state to find the optimal policy for maximizing the cumulative reward.

### 2.3. RL Methods

Several researchers have studied methods to learn optimal policy. Among them, deep Q-learning network (DQN) is a value-based RL that learns an optimal policy by predicting the expected value of reward through state and action pairs [15]. The DQN has the advantage of learning by comparing the expected value of action in the environment, even if no prior knowledge exists. However, the DQN has a limitation because it overestimates the expected value of action. A double DQN was proposed to alleviate the overestimation problem [16]. Compared to the DQN, the double DQN uses an online network and a target network to evaluate the current state and the next state. The policy-based RL directly approximates the policy that determines the agent’s behavior. This has the advantage that the learning convergence is better than the value-based RL, because it is trained to maximize the defined objective function. However, policy-based RL is limited because the learning convergence speed is slow because it is challenging to determine whether the optimal action is selected. An actor–critic method was proposed to complement the advantages and disadvantages of value-based and policy-based RL [17]. The actor–critic method comprises an actor network and a critic network. The critic network estimates the value of the state–action pair. In contrast, the actor network determines the action using a given state. In addition, the critic network adjusts the action according to the evaluated value of the state–action pair by the critic network.

### 2.4. Edge Collaboration for Computation Offloading

Various computation offloading schemes have been proposed to determine the offloading target based on heuristic methods. J. Liu et al. [18] modeled the queuing delay of a task by implementing a Markov chain to reduce the energy consumption of the IoT device. H. Tan et al. [19] proposed the OnDisc algorithm by analyzing the task creation and processing in the real environment to reduce the service time and energy consumption of the task. X. Deng et al. [20] proposed a cloudlet assisted cooperative task assignment (CACTA) system based on the exponential moving average (EMA) prediction to reduce the processing costs in mobile edge computing environments. Y. Zhang et al. [21] used a wholesale and buyback model for improving the utilization costs of computational resources. The latency-classification-based deadline-aware (LCDA) scheme [22] handles target offloading differently, depending on the characteristic of the task. However, this scheme does not provide sufficient computing resources for delay-sensitive tasks. Thus, the scheme is limited because the service times of delay-intensive tasks are increased. H. Zhao et al. [23] scheduled a task based on the greedy algorithm to improve the QoE. X. Xu et al. [24] proposed a heuristic offloading method (HOM) algorithm to reduce the transmission delay of computation offloading based on the exploration of offloading distance. Despite low transmission delays, HOM exhibited high service times due to the limited computing resources. A. Jonathan et al. [25] localized the edge server location to select the offloading target for processing and transmission tasks. W. Zhuang et al. [26] solved a mixed-integer nonlinear program problem formulated to satisfy the task delay requirement, and reduced device battery consumption. X. Chen et al. [27] scheduled a task based on game theory to solve multi-user computation offloading. M. Du et al. [28] tackled the NP-hard problem on the basis of the collaboration between computing nodes based on the min-cut algorithm to improve utilization of network and computing resources.

### 2.5. RL-Based Computation Offloading

According to the system model, the action of the edge server is mutually influenced. For example, the edge server decides and sends a task to the collaboration target, thus affecting the edge server’s computing resources, network bandwidth, and buffer. With this sequential decision-making in a distributed edge environment, computation offloading can be modeled as a Markov decision process (MDP), and an optimal policy can be found using RL. Some states exist with infinite continuous values, such as computing resources and network bandwidth. Moreover, since conventional RL is limited in handling extremely large or infinite state spaces, we used deep neural network-based DRL to handle infinite states [29,30].

Ong et al. [31] made the first attempt to empower thousands of machines. Mnih et al. [32] and Barth-Maron et al. [33] proposed the asynchronous advantage actor–critic (A3C) algorithm and the distributed distributional deterministic deep policy gradient (D4PG) algorithm, respectively, which are the state-of-the-art distributed versions of policy gradient-based DRL algorithms. X. Qiu et al. [7] proposed the distributed and collective DRL-based algorithm (DC-DRL), using n-step learning and experience sharing.

In the existing distributed environment, training DRL requires several CPUs and GPUs. Therefore, distributed DRL in a computation offloading environment has limitations. For example, high communication overhead occurs in RL using A3C because of the sharing parameters between agents. The D4PG algorithm shares the gradient between agents to learn the optimal policy. In particular, gradient sharing between agents has the advantages of small size and high stability compared to parameter sharing. However, the gradient is shared periodically, increasing the communication overhead. The DC-DRL [7] algorithm has high robustness and scalability compared to sharing parameters or gradients, because experiences are shared between agents. However, this approach exhibits low learning efficiency, resulting from unnecessary experience sharing. J. Chen et al. [34] selected an offloading target based on DRL to minimize task service times. In particular, they used a conv1D layer and long-short-term memory (LSTM) instead of an FC layer to improve the convergence of learning. B. Gu et al. [35] used A3C to reduce the service times of tasks and the battery consumption of devices. A3C allows an agent to experience various environments in parallel to achieve high rewards. B. Yamansavascilar et al. [36] proposed a delayed action to handle the effect of the agent’s action that was not immediately obtained in computation offloading environments. However, the immediate reward was also affected by other actions; thus, the reward could not be maximized. L. Ale et al. [37] proposed the Dirichlet deep deterministic policy (D3PG) to minimize the service times of tasks. They improved the performance by adding noise to the action, based on the Ornstein–Uhlenbeck process in the DDPG structure. A. Oadeer et al. [38] used conv1D layer and gated recurrent unit (GRU) to extract temporal features, but feature extraction using a recurrent neural network (RNN) slowed down learning due to sequential data input.

Table 1 shows a summary of recent offloading schemes. In this study, unlike existing schemes, we extracted features using the state obtained from the environment, and the temporal state obtained using LASSO regression and the attention layer. Moreover, we used two attributes to determine the importance of experience. Accordingly, we sampled experiences to be used, and determined the optimal to improve the DRL ward and convergence time. In the reply buffer of the edge server, useful experiences were shared with other edge servers to solve the problem of data sparsity in learning.

## 3. Proposed Scheme

This section represents the DRL-based computation offloading algorithm. Firstly, we introduce a task service time and load balance model. Secondly, we focus on the DDPG framework for reward maximization. Finally, we describe experience sharing to reduce the data sparsity problem.

### 3.1. System Model

**Figure 1:**
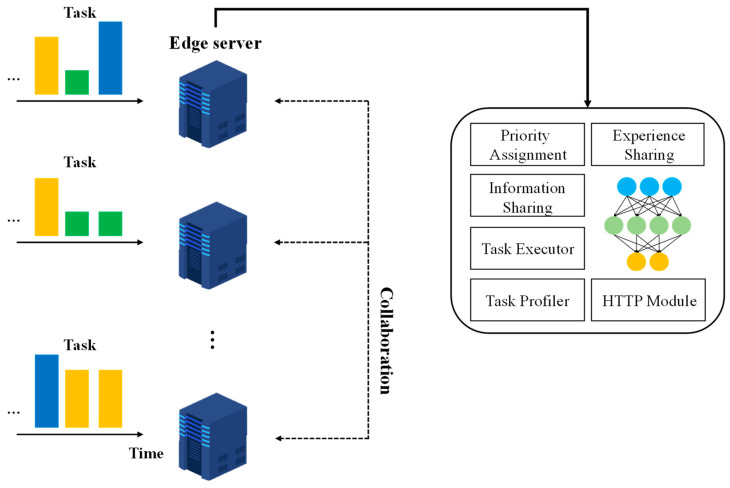
The structure of the proposed scheme.

Figure 1 shows the structure of the proposed scheme. The devices create tasks of different sizes to deadlines, and send them to the local edge server. The edge server collects the size and deadline information for each task, using the task profiler module. The edge server offloads and receives a task from the HTTP module, and processes the assigned task in the task executor module. In the information sharing module, the edge server shares the number of tasks to be processed and the network bandwidth between edge servers. The edge server uses the shared information to select an offloading target to process the task, based on RL in each time slot. The time slot is a few milliseconds. This study was based on certain assumptions. Firstly, we assumed that the edge server schedules tasks based on the first-in, first-out (FIFO) principle. Secondly, the edge server periodically interacts to recognize the state of other edge servers. Thirdly, the number of tasks that the edge server can process simultaneously is limited. Finally, the device does not have sufficient computing resources to process a task within the deadline. Therefore, the device sends a task to the edge server whenever a task is created. This is a multi-agent scenario where each edge server has one agent. The agent in each edge server actions by extracting features based on LAFE to determine the optimal collaboration target. Whenever an agent decides to offload, it stores its current state, action, reward, and the next state in the buffer. We computed the rank of the experiences stored in the buffer, and fetched the experiences according to their rank. However, if the number of devices connected to the edge server is small, collaborative decisions do not occur frequently. This implies that the difference in losses between agents (called “policy gap” [39]) is significant, because agents do not have enough experience to improve their rewards. To solve this problem, we shared experiences between edge servers according to the strategy gradient.

### 3.2. Problem Formulation

We modeled each element of the objective function to minimize the task service time and maximize the load balance between edge servers. The task service time consists of the processing, transmission, and waiting times. The edge server allocates the computing resources to the tasks that need to be processed. The task processing time of a task is calculated using Equation (3).

(3) $$T_{j}^{pr}=\frac{L_{j}\cdot x_{i}}{C_{i}^{max}}$$

$L_{j}$ denotes the size of the task, $x_{i}$ denotes the number of tasks being processed by the edge server i, j denotes the index of the task, i denotes the index of the edge server, and $C_{i}^{max}$ denotes the computing resources capacity of the edge server. If the edge server does not have tasks that are being processed, it allocates all available computing resources to the task. Moreover, the edge server allocates the computing resources according to the characteristics of the tasks. The transmission time of the task is calculated using Equation (4).

(4) $$T_{j}^{tr}=\frac{L_{j}}{ABW_{i,i^{\prime }}}$$

$ABW_{i,i^{\prime }}$ denotes the available bandwidth between the edge servers. Since the transmission time is affected by the network bandwidth between the edge servers, transmission delays occur depending on the network link sharing, even if the collaboration target for each task differs. The tasks sent from the device and edge server are stored in the buffer. Subsequently, the tasks are processed sequentially according to the state of the edge server. The waiting time of the task is calculated using Equation (5).

(5) $$T_{j}^{wait}=\frac{x_{i}\cdot \sum_{j^{\prime }=1+x_{i}}^{\left|J_{i}^{\prime }\right|}L_{j^{\prime }}}{C_{i}^{max}}$$

$J_{i}^{\prime }$ denotes the set of tasks stored in the edge server’s buffer, and $j_{i}^{\prime }$ denotes the index of the task stored in the edge server’s buffer. The waiting time represents the delay until the offloaded task is allocated from the edge server. Since the edge server allocates computing resources according to characteristics of the tasks, the waiting time increases as the number of tasks being processed increases. Based on Equations (3)–(5), the service time of a task is modeled as Equation (6).

(6) $$T_{j}=T_{j}^{pr}+T_{j}^{tr}+T_{j}^{wait}$$

The QoE is affected by the service time of the task and the load balancing between edge servers. We defined the load of the edge server as the number of tasks processed by the edge server, and the number of tasks stored in the buffer. Therefore, the load on the edge server can be expressed using Equation (7).

(7) $$\ell_{i}=x_{i}+\left|J_{i}^{\prime }\right|$$

Various methods for measuring load balance have been proposed. In particular, Jain’s fairness index [40] was used for resource balancing measures in previous studies [41,42]. Therefore, we utilized Jain’s fairness index to model the load balance between edge servers as Equation (8).

(8) $$J=\frac{{\left(\sum_{i=1}^{\left|E\right|}\ell_{i}\right)}^{2}}{\left|E\right|\cdot \sum_{i=1}^{\left|E\right|}\ell_{i}^{2}}$$

E denotes the set of edge servers. Based on the service time of the modeled task and the load balance between the edge servers, the objective function can be defined using Equation (9).

(9) $$R=\max\left\{\alpha J-\beta T_{j}\right\}\;$$

(10) $$s.t.\;C_{i}^{max}\geq c_{i}$$

(11) $$x_{i}^{max}\geq x_{i}$$

(12) 0≤α,β≤1

α denotes the weight of the load balance between edge servers, β denotes the weight of the service time of the task, $c_{i}$ denotes the computing resources used by edge servers, and $x_{i}^{max}$ denotes the maximum number of tasks that the edge server can process. Constraint (10) implies that the computing resources allocated to a task do not exceed the computing resource capacity of the edge server. Constraint (11) indicates that the number of processing tasks at the edge is lower than that of the maximum processing tasks. Constraint (12) indicates that the load balance and service time weights are between 0 and 1. In this research, we determined the collaboration target based on the deep deterministic policy gradient (DDPG) to minimize the task service time and maximize the load balance between edge servers.

### 3.3. Deep Reinforcement Learning Structure

The DDPG generally comprises a state, an action, and a reward. The agent decides on the action based on the state obtained from the environment. The agent affects the environment through the action, and the reward and next state are returned according to the effect of the action. We defined the state, action, and reward as follows:

- State: Task size ($L_{j}$), task deadline ($D_{j}$), set of computing resource capacity of edge server ($C^{max}=\left\{C_{1}^{max},C_{2}^{max},\cdots ,C_{i}^{max}\right\}$), set of computing resource usage $\left(c=\left\{c_{1},c_{2},\cdots ,c_{i}\right\}\right)$, set of available bandwidth between edge servers ($ABW=\left\{ABW_{1},ABW_{2},\cdots ,ABW_{i}\right\}$), and a set of number of stored tasks in edge server’s buffer ($J^{\prime }=\left\{J_{1}^{\prime },J_{2}^{\prime },\cdots ,J_{i}^{\prime }\right\}$).
- Action: The action represents the offloading target. The offloading target is the local edge server or other edge servers.
- Reward: The reward is defined by considering task service time and load balance, as calculated in Equation (9).

**Figure 2:**
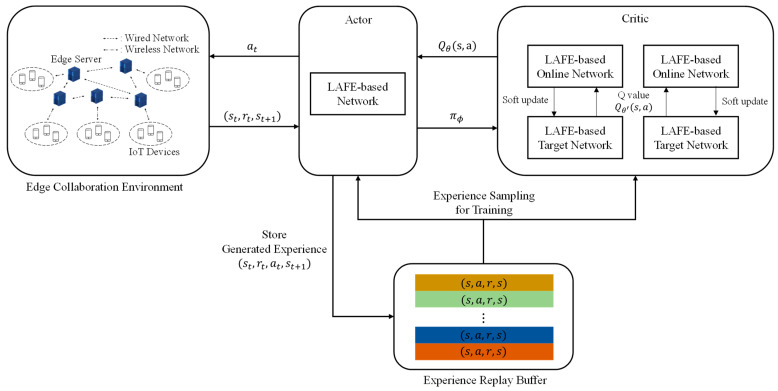
The proposed DDPG structure.

In this study, the set of states is determined using the number of edge servers and network links connected between edge servers. However, some states, such as computing resource usage and the available bandwidth, have infinite continuous values within the range. Therefore, we deployed DRL, which can handle huge state spaces. After defining the state, action, and reward, the proposed DDPG was trained. Figure 2 shows the proposed DDPG structure. We selected an action using the current state obtained from the environment based on the actor–critic method. The actor–critic approach consists of an actor network and a critic network. The actor network uses the state of the environment to determine the agent’s action. Therefore, the actor network obtains the state from the distributed edge environment, and determines the collaboration target using the LAFE-based network. The edge server sends the task to the collaboration target, and obtains the results of the processing task and the next state of the environment. The actor network stores $\left(s_{t},a_{t},r_{t},s_{t+1}\right)$ in the experience replay buffer. Moreover, the actor–critic algorithm learns the policy after each episode. The proposed DDPG uses several $\left(s_{t},a_{t},r_{t},s_{t+1}\right)$ that are sampled according to the rank of experience in the replay buffer. It updates the parameters of the actor network after updating the parameters of the critic network. The critic network evaluates the state and action pair determined by the actor network. In addition, in the critic network, the online network and target network are used to alleviate overestimating the value. We used a two-pair network to reduce the variation in the value evaluated by the critic network. In the critic network, the parameters of the online network are updated using the difference between the values estimated by the two networks. Unlike the existing DDPG algorithm, we only used one network in the actor network for learning efficiency through experience generated by other agents. Subsequently, DDPG updates the parameters of the actor network using the Q values. The target network of the critic network softly updates the parameters.

**Figure 3:**
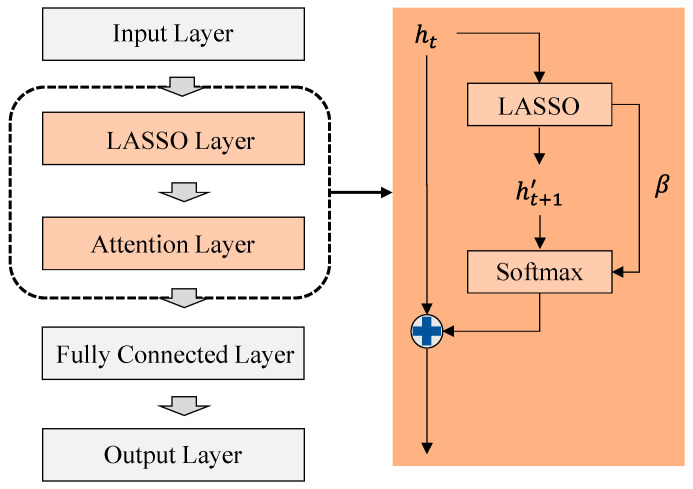
The structure of LAFE.

Figure 3 shows the structure of LAFE. LAFE consists of LASSO regression and attention layers. The LASSO layer extracts temporal features by predicting the next state. Since this output shows features for the next state, we connected the output of the input layer to the output of the LASSO layer. LASSO reduces the weight of features that have little influence on the output when learning with LAFE; however, some unnecessary features remain. Therefore, we used the attention layer to reduce the feature weight to improve performance. The temporal state can be predicted using Equation (13).

(13) $$h_{t,x}^{\prime }=h_{t,1}\beta_{1}+h_{t,2}\beta_{2}+\cdots +h_{t,x}\beta_{x}$$

$h_{x}$ denotes the feature from the previous layer, and $\beta_{x}$ denotes the regression parameter. These predicted and extracted features are concatenated according to the state in Equation (14).

(14) $$\hat{h_{t}}=h_{t}⊕h_{t}^{\prime }$$

LASSO updates the weights using the L1-norm. Therefore, when several features are used for prediction, LASSO reduces the effect of unnecessary features by shrinking the weight of each feature [43]. Moreover, LASSO regularizes the prediction model as Equation (15).

(15) $$\min\{\overset{T}{\underset{t=1}{\sum }}{\left(h_{t}^{\prime }-\underset{x=1}{\sum }h_{t,x}\beta_{x}\right)}^{2}+\lambda \left|\beta \right|\}$$

λ denotes the shrinkage parameter of LASSO. Although LASSO reduces input noise, features with small values affect the output. We used the attention layer to focus on the important features. The attention weight for parameter updating of the regression is calculated as Equation (16).

(16) $$a_{t}=\sigma \left(h_{t}^{T}\cdot \beta \right)$$

σ denotes the sigmoid function, and $h_{t}^{T}$ denotes the transpose of the feature vector extracted from linear regression. In particular, the attention weight has a lower value when the feature weight of the regression is small. In other words, we only considered important features by reducing their weight. The regression weight according to the attention weight is calculated as Equation (17).

(17) $$\beta^{\prime }=\sum_{x=1}^{X}a_{t,x}\cdot \beta$$

The proposed DDPG structure determines the action, and evaluates the state using LAFE. In addition, this structure stores the current state, action, reward, and next state in the buffer, and samples and fetches the experiences according to the importance of learning. We considered the loss of the critic network and the TD error to determine the importance of experience. The TD error is calculated as Equation (18).

(18) $$\delta_{t}=R_{t+1}+\gamma V\left(s_{t+1}\right)-V\left(s_{t}\right)$$

$R_{t}$ denotes the reward, γ denotes the discount factor, and $V\left(s_{t}\right)$ denotes the state value. The TD error indicates the difference between the estimated and predicted values for the next state. A high TD error means that the loss of the critic network is high. Therefore, DRL can improve the reward using experience with a high TD error. However, the high-rank experience generated when the gradient is high is sufficiently used in the early learning episodes. Therefore, the learning effect is low in the late episode of learning. For a high learning reward, even if the TD error of the two experiences is the same, the importance is set differently according to the loss. We calculated the rank of the experience as Equation (19).

(19) $$r=\frac{\left|\delta_{t}\right|}{L}+\left|\delta_{t}\right|+L$$

L denotes the loss of critic network. Moreover, the experiences can have the same importance by calculating the rank according to the TD error and learning progress. This is problematic in experiences with high TD errors that are not frequently used. Thus, we used a bias term with TD error and loss to treat the importance differently to solve this problem. Based on the calculated experience rank, the probability of experience is calculated as Equation (20).

(20) $$p=\frac{r_{\varphi }+\left|r_{min}\right|}{\sum_{\varphi =1}^{\left|\Phi \right|}\left(r_{\varphi }+\left|r_{min}\right|\right)}$$

φ denotes the index of experience stored in the buffer, Φ denotes the set of experiences stored in buffer, and $r_{min}$ denotes the minimum rank value in experiences. In general, high rank experiences are used for learning. However, when it is frequently used for learning, the learning bias is increased. The bias increase can be handled by calculating the weight for network parameter updating, as in Equation (21) [44].

(21) $$\omega =\frac{1}{p^{\ell }}$$

ℓ denotes the annealing weight. As the fetching probability of experience is increased, the weight is decreased to reduce the bias. According to this weight, the network parameters can be updated using Equations (22) and (23).

(22) $$\theta \leftarrow \theta +\omega \nabla_{\theta }Q\left(s_{t},a_{t}\right)$$

(23) $$\phi \leftarrow \phi +\omega \nabla_{\phi }J$$

**Figure 4:**
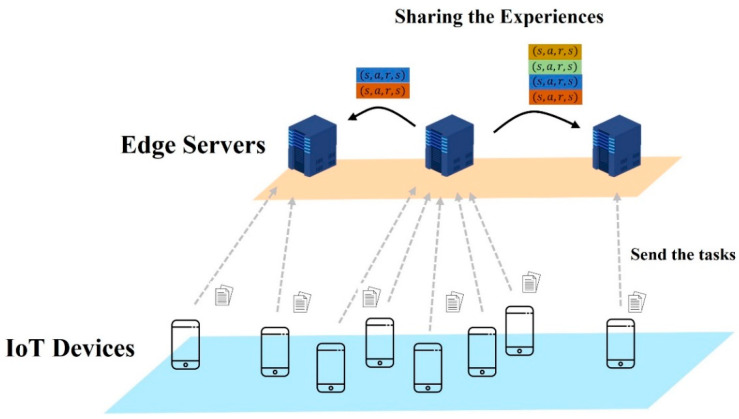
Example of experience sharing between agents.

$\nabla_{\phi }J$ denotes the strategy gradient. The parameters of the target network are softly updated according to the smoothing parameter, τ. Even if learning is conducted based on LAFE and the importance of experience in a distributed edge environment, the experience required for learning is insufficiently generated when only a few devices are connected to the edge server. Therefore, the agent of an edge server with a small number of connected devices has a high strategy gradient. To reduce the strategy gradient, we shared the experience between agents. Figure 4 shows an example of experience sharing between agents. Note that each edge server connects to a different number of devices, and each device sends a task to the edge server whenever a task is created. The agent on the edge server determines the offloading target whenever it receives the task, and stores the experience in the buffer. The agent verifies the strategy gradient of other agents to determine the experience to be shared. When the number of devices connected to the edge server is small, an agent with a high strategy gradient receives numerous experiences from other agents. In contrast, an agent with a low strategy gradient has fewer shared experiences. The experience shared between agents can be determined through Equation (24).

(24) $$L_{k}=\{\varphi \in \Phi |r_{\varphi }\rangle \left(\frac{r_{max}^{B}+r_{min}^{B}}{2}\right)\cdot \left(1+\frac{\left|\nabla_{\phi }J_{i}\left|-\right|\nabla_{\phi }J_{k}\right|}{\left|\nabla_{\phi }J_{i}\right|}\right)\}$$

B denotes the size of the mini-batch, $r_{max}^{B}$ denotes the maximum rank value in the mini-batch, and $r_{min}^{B}$ denotes the minimum rank value in the mini-batch. The agents share experiences adaptively according to the strategy gradient. When the strategy gradient is the same, the agents share half of their experiences in the buffer. Algorithm 1 shows the pseudocode of the DDPG with prioritized experience sharing. $M_{max}\;$denotes the maximum number of episodes, and Z denotes the number of epochs. Each edge server contains a different agent, and each agent makes an independent offloading decision. For learning the policy, several episodes are used. In an episode, RL initializes the state and reward to zero to start learning. At the beginning of learning, the action of the edge server is determined using the state obtained from the environment based on the randomly generated policy during the simulation time. Depending on the action, the edge server obtains the reward resulting from the action and the next state of the environment. The edge server stores the current state, action, reward, and next state paired with its TD error and the state value in the experience replay buffer. The replay buffer calculates its rank based on Equation (19), whenever experience is stored. The replay buffer removes the less used experience when the buffer is full. When the simulation finishes, RL samples the experience from the replay buffer and iteratively proceeds with the learning process. RL updates the parameters of the actor network and the online network of critic, using weights calculated based on the rank of experience. After the parameters of these networks are adjusted, the critic’s target network is softly updated to improve the stability of learning. When training finishes, RL shares useful learning experiences with other edge servers and performs the next episode.
**Algorithm 1** RL with Prioritized Experience Sharing1: 2: 3: 4: 5: 6: 7: 8: 9: 10: 11: 12: 13: 14: 15: 16: 17: 18: 19: 20: 21: 22: 23: **Initialize** DDPG networks $Q_{\theta_{1}}$$,\;Q_{\theta_{2}}$, actor network $\pi_{\phi }$ with random parameters ϕ, and random parameters in critic network $\theta_{1}$$,\theta_{2}$. **Initialize** target network parameter $\theta_{1}^{\prime },\theta_{2}^{\prime }$ **for** episode = 1 **to** M_max_
**do**
  Reset environment state s_0_ and reward r = 0   **for** t = 1 **to** T **do**   Obtain the state s_t_   Generate action a_t_ by the actor network   Obtain reward r_t_ and state s_(t+1)_ using shared information between edge servers    Store the experience $\varphi_{t}=\left(s_{t},{\;a}_{t},r_{t},s_{t+1}\right)$ with TD error and state value   Calculate the rank according to the Equation (9)   **if** replay buffer is full **then**     Find the least replayed experience $\varphi^{\prime }$ in replay buffer     Remove $\varphi^{\prime }$ from replay buffer   **end if**  **end for**   **for**
j=1 **to** Z **do**   Probabilistically select a sample from replay buffer   Calculate ω according to the Equation (11)   Update online network parameter of critic and actor   Soft update critic network parameter of critic and actor  **end for**  Share the prioritized experience **end for**

## 4. Simulation

### 4.1. Simulation Setup

To evaluate the performance of the proposed scheme, we used the Keras framework [45], and created a task on the basis of the Poisson distribution. The number of edge servers was set to 5, the computing resources of the edge server were set to 10000 MIPS, the bandwidth between edge servers was set to 50 Mbps, the number of devices was set to 200, $x^{max}$ was set to 3, α was set to 0.7, β was set to 1, and λ was set to 0.7. The proposed scheme was compared with *DDPG*, *DDPG-PER*, *DC-DRL*, and *TADPG*. *DDPG* learns the optimal policy of task offloading using the replay buffer. *DDPG-PER* learns the policy with a prioritized experience. The priority of experience is determined by the TD error. *DC-DRL* trains the computation offloading policy using n-step learning and experience sharing. However, *DC-DRL* fetches the experience regardless of its importance. *TADPG* [34] learns the optimal policy using conv1D and LSTM.

The learning rates of the actor network and critic network were set as 0.0001 and 0.001, respectively. The number of layers and the number of neurons in the hidden layer were the same in both networks. The number of layers was set to 4, and the number of neurons was set to 32. The buffer size was set to $10^{5}$, the discount factor was set to 0.9, and the fetch sample size was set to 16. We used the softmax function in the output layer of the actor network. When reinforcement learning is trained, actions are randomly selected for exploration. When we tested the learned model, an action with a high probability was selected.

### 4.2. Performance of RL Training

**Figure 5:**
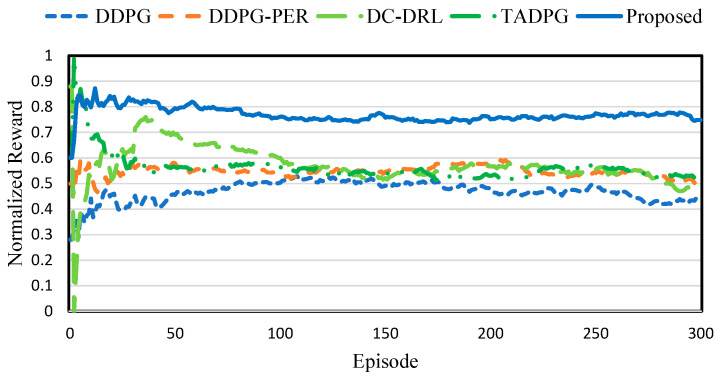
Normalized reward comparison.

Figure 5 shows the normalized reward comparison. *DDPG* exhibited lower reward than the other schemes. This is because *DDPG* learns the offloading policy using randomly selected experiences. Moreover, this scheme does not improve other edge servers’ RL policy because it only uses locally generated experiences. *DDPG-PER* utilizes the importance of experience according to the TD error. The high importance of experience has a high probability for fetching. Therefore, *DDPG-PER* exhibited higher reward than *DDPG*. In addition, *DC-DRL* basically learns the policy similar to *DDPG*. However, unlike *DDPG*, *DC-DRL* shares the experiences between agents. However, DC-DRL exhibited a lower performance than *DDPG-PER*. This is because *DC-DRL* utilized the sampled experiences that were not useful. *TADPG* learns the policy based on the temporal state and prioritized experience. The prioritized experience is calculated using the TD error and policy gradient. In particular, *TADPG* extracts the temporal features using the conv1D and LSTM layers for training efficiency. However, in *TADPG*, the edge server to which a small number of devices is connected does not sufficiently learn the agent. Furthermore, *TADPG* only uses the temporal state to determine the action. It provided similar performance to *DDPG- PER* and *DC-DRL*. The proposed scheme trains the offloading policy on the basis of the LASSO regression. In particular, the proposed scheme uses the prioritized experiences according to the TD error and training loss, and adaptively shares experience according to the strategy gradient. In the simulation results, the proposed scheme achieved a higher reward than the other schemes, according to the useful experience shared and sampled. Table 2 shows the reward comparison and variation. The proposed scheme achieved a 12.5% higher reward than *TADPG*. It also achieved a 47.6% lower reward variation than *DDPG*.

**Figure 6:**
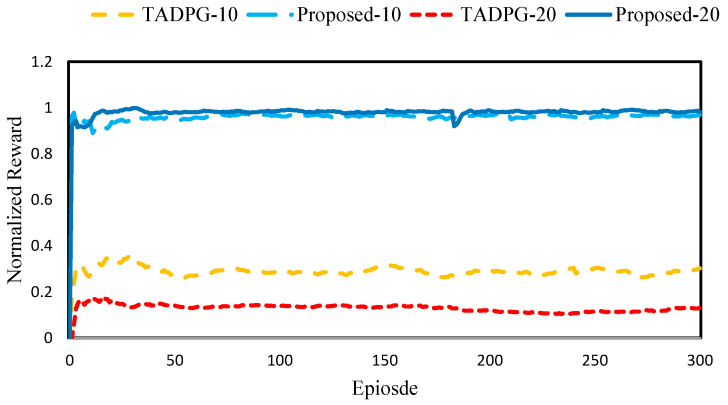
Reward variation comparison.

Figure 6 shows the reward variation comparison. In particular, *DDPG* and *DDPG-PER* indicate that the reward variations are similar. This is because these schemes only used the experience generated by the local edge server. Therefore, there was a significant policy gap in these schemes because the edge servers, where few tasks are generated, provided insufficient learning. *TADPG* determines an action using LSTM, and can achieve a high reward based on the importance of experience. However, this scheme exhibited similar performance to *DDPG* and *DDPG-PER* due to the difference in the number of experiences available for learning in each edge server. In addition, *DC-DRL* exhibited a performance that was similar to other existing schemes, despite the experience sharing. This is because learning through *DC-DRL* is inefficient for learning, even if it shares a sufficient experience with other edge servers. Thus, *DC-DRL* cannot improve the accuracy of the state value and reduce the loss of networks. The proposed scheme selects the experience to share based on the strategy gradient of other agents. In particular, an agent with a high strategy gradient can sufficiently learn by sharing several experiences. The results, therefore, indicate that the proposed scheme outperformed the existing schemes regarding reward variation.

**Figure 7:**
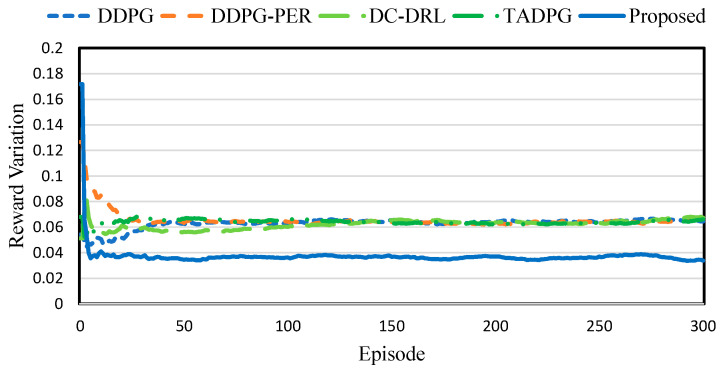
Normalized reward comparison with 10 and 20 edge servers.

Figure 7 shows the normalized reward comparison with 10 and 20 edge servers. Similar to the case of 5 edge servers, the proposed scheme consistently showed better performance than *TADPG* in the cases of 10 and 20 edge servers. As the number of edge servers increased, simulations took a lot of time, since each edge server performed the reinforcement learning for the offloading decision. Table 3 shows the reward comparison with 10 and 20 edge servers. *Proposed-20* achieved a 28.7% higher reward than *TADPG-20*. *Proposed-10* achieved a 55.1% higher reward than *TADPG-10*. Therefore, applying the proposed scheme to a larger scale edge computing environment is left for future research.

### 4.3. Performance of QoE

**Figure 8:**
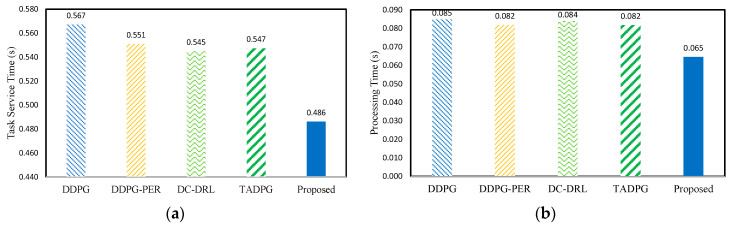

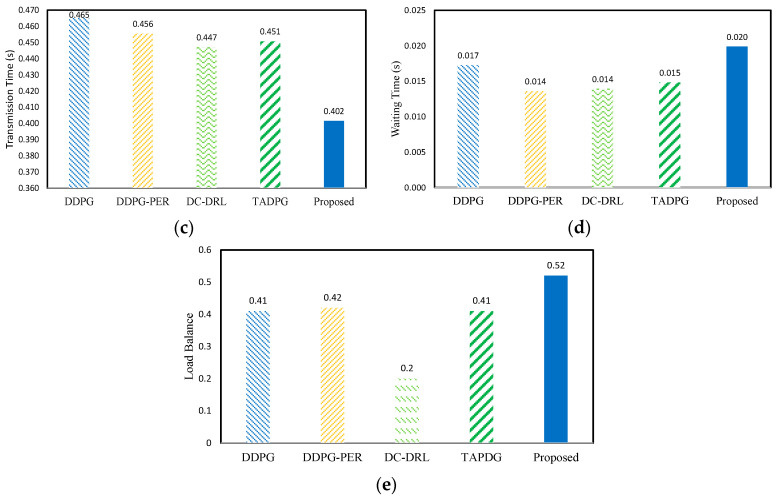
QoE comparison. (**a**) Task service time; (**b**) task processing time; (**c**) task transmission time; (**d**) task waiting time; (**e**) load balance.

Figure 8 shows the QoE comparison. *DDPG* uses a randomly sampled experience for learning. Therefore, *DDPG* exhibited a high task service time. *DDPG-PER* and *TADPG* showed a similar load balance performance. Note that *DC-DRL* trains the policy based on randomly shared and selected experiences. If the reward in experience is high due to low task service time, the agent shows a high bias for one of several objectives. Therefore, *DC-DRL* achieved lower service time than *DDPG*, even though the load balance was low. This is because the RL agent did not focus on each objective of the reward when learning. In other words, the RL agent maximizes the cumulative reward, but this does not guarantee that it maximizes the performance for each objective of the reward. The proposed scheme learns the optimal policy based on LASSO regression, prioritized experience, and adaptively sharing the experience. As a result, the QoE of the proposed scheme outperformed those of the existing schemes.

## 5. Conclusions

Edge computing is an emerging computing paradigm that encompasses several networks and servers near the user or the data source. Various researchers have proposed collaboration methodologies between edge servers to improve the efficiency of task processing based on various offloading schemes.

In this study, we introduced a distributed DRL-based computation offloading scheme for improving the QoE in edge computing environments. The proposed scheme models the load balance between the edge servers and the task service time for the DRL reward. The proposed approach has three contributions. Firstly, LASSO predicts the next state by lowering the importance of unnecessary data for prediction among the input data. Since LASSO does not remove unnecessary data, the prediction accuracy is low. To improve the accuracy, we used an attention layer. The attention layer re-ranked the importance of the data. Secondly, when training the DRL, we prioritized experiences according to the loss of the critic network, as well as the TD error, to improve the efficiency of learning according to the training progress. Finally, the proposed scheme shared experience based on the strategy gradient between agents to handle the data sparsity problem. In particular, the proposed scheme transmitted high-priority experiences in the replay buffer so that shared experiences could improve the agent’s performance The proposed scheme also adjusted the number of shared experiences by considering the agent’s strategy gradient, in order to reduce the load on the network. Simulation results showed that the proposed scheme has higher reward and lower reward variations than existing schemes in multi-agent scenarios. The proposed scheme also reduces the policy gap through sharing experiences. However, since the proposed scheme adjusts parameters of the reinforcement learning model using a fixed learning rate, there is a problem that the policy gap still exists.

In future research, we will apply the proposed scheme to a large-scale edge computing environment, in order to check its scalability and investigate the online training methodology to handle changing sets of states adaptively. In addition, we will implement the proposed scheme to evaluate its performance in a real environment.

## Figures and Tables

**Table 1:** Summary of recent offloading schemes.

Scheme	Temporal State	Experience Priority	Agent
TADPG [34]	✓	✓	Single-agent
B. Gu [35]	✕	✕	Multi-agent
DC-DRL [7]	✕	✕	Multi-agent
L. Ale et al. [37]	✕	✕	Single-agent
A. Oadder et al. [38]	✓	✓	Single-agent
Proposed	✓	✓	Multi-agent

**Table 2:** Reward and variation comparison.

	Proposed	TADPG	DC-DRL	DDPG-PER	DDPG
Reward	−0.44977	−0.51398	−0.53132	−0.5213	−0.53887
Variation	0.0338	0.0661	0.0673	0.0655	0.0645

**Table 3:** Reward and variation comparison.

	Proposed-20	TADPG-20	Proposed-10	TADPG-10
Reward	−0.0523	−0.0733	0.0242	0.0156

## Data Availability

Not applicable.

## References

[1] Aslanpour M.S., Gill S.S., Toosi A.N. (2020). Performance Evaluation Metrics for Cloud, Fog, and Edge Computing: A Review, Taxonomy, Benchmarks, and Standards for Future Research. Internet Things.

[2] Khan W.Z., Ahmed E., Hakak S., Yaqqob I., Ahmed A. (2019). Edge Computing: A Survey. Future Gener. Comput. Syst..

[3] Hu Y., Zhan J., Zhou G., Chen A., Cai W., Guo K., Hu Y., Li L. (2020). Fast Forest Fire Smoke Detection using MVMNet. Knowl.-Based Syst..

[4] Wang J., Pan J., Esposito F., Calyam P., Yang Z., Mohapatra P. (2020). Edge Cloud Offloading Algorithms: Issues, Methods, and Perspectives. ACM Comput. Surv..

[5] Meng Q., Wang K., Liu B., Miyazaki T., He X. (2018). QoE-based Big Data Analysis with Deep Learning in Pervasive Edge Environment. Proceedings of the IEEE International Conference on Communications.

[6] Xue M., Wu H., Peng G., Wolter K. (2021). DDPQN: An Efficient DNN Offloading Strategy in Local-Edge-Cloud Collaborative Environments. IEEE Trans. Serv. Comput..

[7] Qiu X., Zhang W., Chen W., Zhen Z. (2020). Distributed and Collective Deep Reinforcement Learning for Computation Offloading: A Practical Perspective. IEEE Trans. Parallel Distrib. Syst..

[8] Li Y. (2017). Deep Reinforcement Learning: An Overview. arXiv.

[9] Lillicrap T.P., Hung J.J., Pritzel A., Heess H., Erez T., Tassa Y., Silver D., Wierstra D. (2015). Continuous Control with Deep Reinforcement Learning. arXiv.

[10] Satyanarayann M. (2001). Pervasive Computing: Vision and Challenges. IEEE Pers. Commun..

[11] Song H., Bai J., Yi Y., Wu J., Liu L. (2020). Artificial Intelligence Enabled Internet of Things: Network Architecture and Spectrum Access. IEEE Comput. Intell. Mag..

[12] Bonomi F., Milito R., Zhu J., Addepalli S. (2012). Fog Computing and Its Role in the Internet of Things. International Conference on Mobile Cloud Computing Workshop.

[13] Patal M., Hu Y., Hede P., Joubert J., Thornton C., Naughton B., Ramos J.R., Chan C., Young V., Tan S.J. (2013). Mobile-Edge Computing-Introductory Technical White Paper. ETSI Tech. Represent..

[14] MCEnros P., Wang S., Liyanage M. (2022). A Survey on the Convergence of Edge Computing and AI for UAVs: Opportunities and Challenges. IEEE Internet Things J..

[15] Mnih V., Kavukcuoglu K., Silver D., Graves A., Antonoglou I., Wierstra D., Riedmiller M. (2013). Playing Atari with Deep Reinforcement Learning. arXiv.

[16] Van Hasselt H., Guez A., Silver D. (2016). Deep Reinforcement Learning with Double Q-Learning. AAAI Conf. Artif. Intell..

[17] Konda V., Tsitsiklis J. (1999). Actor-Critic Algorithms. Advances in Neural Information Processing Systems 12.

[18] Liu J., Mao Y., Zhang J., Letaief K.B. (2016). Delay-Optimal Computation Task Scheduling for Mobile-Edge Computing Systems. IEEE International Symposium on Information Theory.

[19] Tan H., Han Z., Li X.-Y., Lau F.C. (2017). Online Job Dispatching and Scheduling in Edge-Clouds. IEEE Conference on Computer Communications.

[20] Deng X., Li J., Liu E., Zhang H. (2020). Task Allocation Algorithm and Optimization Model on Edge Collaboration. J. Syst. Archit..

[21] Zhang Y., Lan X., Ren J., Cai L. (2020). Efficient Computing Resource Sharing for Mobile Edge-Cloud Computing Networks. IEEE/ACM Trans. Netw..

[22] Choi H., Yu H., Lee E. (2019). Latency-Classification-based Deadline-aware Task Offloading Algorithm in Mobile Edge Computing Environments. Appl. Sci..

[23] Zhao H., Wang Y., Sun R. Task Proactive Caching based Computation Offloading and Resource Allocation in Mobile-Edge Computing Systems. Proceedings of the IEEE International Wireless Communications & Mobile Computing Conference.

[24] Xu X., Li D., Dai Z., Li S., Chen X. (2019). A Heuristic Offloading Method for Deep Learning Edge Services in 5G Network. IEEE Access.

[25] Jonathan A., Chandra A., Weissman J. Locality-aware Load Sharing in Mobile Cloud Computing. Proceedings of the International Conference on Utility and Cloud Computing.

[26] Wang Z., Liang W., Huang M., Ma Y. (2018). Delay-Energy Joint Optimization for Task Offloading in Mobile Edge Computing. arXiv.

[27] Chen X., Jiao L., Li W., Fu X. (2015). Efficient Multi-User Computation Offloading for Mobile-Edge Cloud Computing. IEEE/ACM Trans. Netw..

[28] Du M., Wang Y., Ye K., Xu C. (2020). Algorithmics of Cost-Driven Computation Offloading in the Edge-Cloud Environment. IEEE Trans. Netw..

[29] Chen M., Wang T., Zhang S., Liu A. (2021). Deep Reinforcement Learning for Computation Offloading in Mobile Edge Computing Environment. Comput. Commun..

[30] Seid A.M., Boaten G.O., Anokye S., Kwantwi T., Usn G., Liu G. (2021). Collaborative Computation Offloading and Resource Allocation in Multi-UAV-Assisted IoT Networks: A Deep Reinforcement Learning Approach. IEEE Internet Things J..

[31] Ong H.Y., Chavez K., Hong A. (2015). Distributed Deep Q-Learning. arXiv.

[32] Mnih V., Badia A.P., Mirza M., Graves A., Lillicrap T., Harley T., Silver D., Kavukcuoglu K. Asynchrounous Methods for Deep Reinforcement Learning. Proceedings of the 33rd International Conference on International Conference on Machine Learning.

[33] Barth-Maron G., Hoffman M.W., Budden D., Dabney W., Horgan D., Tb D., Muldal A., Hees N., Lillicrap T. (2018). Distributed Distributional Deterministic Policy Gradients. arXiv.

[34] Chen J., Xing H., Xiao Z., Xu L., Tao T. (2021). A DRL Agent for Jointly Optimizing Computation Offloading and Resource Allocation in MEC. IEEE Internet Things J..

[35] Gu B., Alzab M., Lin Z., Hang X., Juang J. (2022). AI-enabled Task Offloading for Improving Quality of Computational Experience in Ultra Dense Networks. ACM Trans. Internet Technol..

[36] Yamansavascilar B., Baktir A.C., Sonmez C., Ozgovde A., Ersov C. (2021). DeepEdge: A Deep Reinforcement Learning based Task Orchestrator for Edge Computing. arXiv.

[37] Ale L., King S.A., Zhang N., Sattar R., Skandaranivam J. (2022). D3PG: Dirichlet DDPG for Task Partitioning and Offloading with Constrained Hybrid Action Space in Mobile Edge Computing. IEEE Internet Things J..

[38] Qadeer A., Lee M.J. DDPG-Edge-Cloud: A Deep-Deterministic Policy Gradient based Multi-Resource Allocation in Edge-Cloud System. Proceedings of the IEEE International Conference on Artificial Intelligence in Information and Communication.

[39] Nachum O., Morouzi M., Xu K., Schuurmans D. (2017). Bridging the Gap Between Value and Policy based Reinforcement Learning. Adv. Neural Inf. Process. Syst..

[40] Jain R.K., Chiu D.-M.W., Hawe W.R. (1984). A Quantitative Measure of Fairness and Discrimination.

[41] Lai S., Fan X., Ye Q., Tan Z., Zhang Y., He X., Nanda P. (2020). FairEdge: A Fairness-Oriented Task Offloading Scheme for IoT Applications in Mobile Cloudlet Networks. IEEE Access.

[42] Zhang G., Shen F., Yang Y., Qian H., Yao W. Fair Task Offloading among Fog Nodes in Fog Computing Networks. Proceedings of the IEEE International Conference on Communications.

[43] Tibshirani R. (2011). Regression Shrinkage and Selection via the LASSO: A Retrospective. J. R. Stat. Soc. Ser. B Stat. Methodol..

[44] Schaul T., Quan J., Antonoglou I., Silver D. (2015). Prioritized Experience Replay. arXiv.

[45] Gulli A., Pa S. (2017). Deep Learning with Keras.

